# CircFBXL5 promotes the 5-FU resistance of breast cancer via modulating miR-216b/HMGA2 axis

**DOI:** 10.1186/s12935-021-02088-3

**Published:** 2021-07-19

**Authors:** Mingzhi Zhu, Yanyan Wang, Fang Wang, Lin Li, Xinguang Qiu

**Affiliations:** 1 Department of Breast Surgery, The First Affiliated Hospital of Zhengzhou University, Jianshe Dong Lu, Erqi District , Zhengzhou City, 450052 Henan Province China; 2 Department of Thyroid Surgery, The First Affiliated Hospital of Zhengzhou University, Jianshe Dong Lu, Erqi District, Zhengzhou City, 450052 Henan Province China

**Keywords:** Breast cancer, 5-Fluorouracil, circFBXL5, HMGA2, miR-216b

## Abstract

**Background:**

Circular RNAs (circRNAs) have been confirmed to be relevant to the 5-fluorouracil (5-FU) resistance of breast cancer. Nevertheless, how and whether circRNA F-box and leucine-rich repeat protein 5 (circFBXL5) regulates the 5-FU resistance of breast cancer is uncertain. This study aims to explore the function and mechanism of circFBXL5 in the 5-FU resistance of breast cancer.

**Methods:**

Thirty nine paired breast cancer and normal tissues were harvested. circFBXL5, microRNA-216b (miR-216b) and high-mobility group AT-hook 2 (HMGA2) abundances were examined via quantitative reverse transcription polymerase chain reaction or western blot. Cell viability, 5-FU resistance, migration, invasion, and apoptosis were tested via cell counting kit-8 assay, wound healing analysis, transwell analysis, and flow cytometry. The relationship of miR-216b and circFBXL5 or HMGA2 was tested via dual-luciferase reporter analysis and RNA pull-down assay. The impact of circFBXL5 on breast cancer tumor growth in vivo was analyzed via xenograft model.

**Results:**

circFBXL5 was highly expressed in breast cancer tissues and cells, and was more upregulated in 5-FU-resistant breast cancer cells. Function experiments showed that circFBXL5 knockdown inhibited the 5-FU resistance of breast cancer by inhibiting cell migration, invasion and promoting apoptosis. In the terms of mechanism, miR-216b could be sponged by circFBXL5, and its inhibitor could also reverse the influence of circFBXL5 silencing on the 5-FU resistance of breast cancer cells. In addition, HMGA2 was a target of miR-216b, and its overexpression also reversed the regulation of miR-216b overexpression on the 5-FU resistance of breast cancer. Furthermore, circFBXL5 interference declined breast cancer tumor growth in xenograft model.

**Conclusion:**

Our data showed that circFBXL5 could promote the 5-FU resistance of breast cancer by regulating miR-216b/HMGA2 axis.

## Introduction

Breast cancer is one common malignant cancer in women [1]. 5-fluorouracil (5-FU) is an antitumor agent via affecting pyrimidine synthesis, which is applied to treatment of solid cancers, including breast cancer [2]. Nevertheless, the resistance of 5-FU limits the efficacy of this drug. Hence, it is necessary to explore new insight into the pathogenesis of 5-fU resistance to breast cancer.

The noncoding RNAs are relevant to various events of breast cancer progression [3]. Circular RNAs (circRNAs) are a group of closed-loop noncoding RNAs without 5′ and 3′ ends [4]. CircRNAs can regulate mRNA expression via competing with microRNAs (miRNAs), thus participating in breast cancer development [5]. Moreover, circRNAs are implicated in regulating the chemoresistance, including 5-FU resistance [6, 7]. For instance, circRNA CDR1as knockdown repressed 5-FU resistance in breast cancer cells via regulating miR-7 [8]. CircRNA F-box and leucine-rich repeat protein 5 (circFBXL5) is a newly discovered circRNA in recent years. Studies had shown that circFBXL5 was associated with the poor prognosis in breast cancer patients, which could facilitate cancer progression via modulating miR-660 [9]. Nevertheless, whether circFBXL5 are involved in the regulation of 5-FU resistance in breast cancer remain unknown.

miRNAs have key roles in tumorigenesis and development of breast cancer [10]. Moreover, the dysregulated miRNAs are associated with the resistance of drugs, including 5-FU [11, 12]. In many cancers, miR-216b could repress the resistance of drugs, such as cisplatin, Adriamycin and 5-FU [13–15]. Moreover, miR-216b could inhibit cell growth and metastasis of breast cancer cells via decreasing syndecan-binding protein (SDCBP) [16]. Nevertheless, the influence of miR-216b on 5-FU resistance to breast cancer is unclear. High-mobility group A (HMGA) proteins play pivotal roles in breast cancer development [17]. HMGA2 is one key member of HMGA family, which is relevant to various cell processes, such as apoptosis, metastasis, and DNA damage repair [18]. The increasing evidences have reported that HMGA2 is associated with the resistance of drugs, including cisplatin, Adriamycin and 5-FU [19–22]. Importantly, HMGA2 is implicated in regulating breast cancer cell growth, invasion and 5-FU resistance mediated via nuclear paraspeckle assembly transcript 1 (NEAT1) [23].

Our study aims to explore the role of circFBXL5 in the 5-FU resistance of breast cancer. Using bioinformatics analysis software, we found that circFBXL5 could complementary bind with miR-216b, and miR-216b could interact with the 3′UTR of HMGA2. Hence, we hypothesized that circFBXL5 might sponge miR-216b to regulate HMGA2, thereby participating in the regulation of 5-FU resistance in breast cancer.

## Materials and methods

### Patients and tissue collection

Thirty nine breast cancer patients who underwent surgery were enrolled from the First Affiliated Hospital of Zhengzhou University. The clinicopathological characteristics of patients were shown in Table 1. The cancer tissues and paired normal tissues were harvested and maintained at − 80 °C. The written informed consent was obtained from all participants. This research was permitted via the ethics committee of the First Affiliated Hospital of Zhengzhou University and conducted under the Helsinki Declaration.

**Table 1:** Correlation between circFBXL5 expression and the clinicopathological features of breast cancer patients

Clinicopathologic features	Relative circFBXL5 level		*P* value
	High (%)	Low (%)	
Age (years)			0.2493
≥ 55	15 (55.6)	12 (44.4)	
< 55	9 (75.0)	3 (25.0)	
Tumor size (cm)			0.0181*
≥ 3	10 (90.9)	1 (9.1)	
< 3	14 (50.0)	14 (50.0)	
TNM stage			0.0052*
I + II	12 (46.2)	14 (53.8)	
III	12 (92.3)	1 (7.7)	
ER status			0.8759
Negative	9 (60.0)	6 (40.0)	
Positive	15 (62.5)	9 (37.5)	
PR status			0.6729
Negative	8 (57.1)	6 (42.9)	
Positive	16 (64.0)	9 (36.0)	
HER2 status			0.2677
Negative	17 (680.0)	8 (32.0)	
Positive	7 (50.0)	7 (50.0)	

^*^P < 0.05

### Cell culture and 5-FU-resistant cells establishment

The breast cancer cell lines MDA-MB-453 and MDA-MB-231 cells were provided via Procell (Wuhan, China) and grown in Leibovitz’s L-15 medium (Thermo Fisher, Waltham, MA, USA) plus 10% fetal bovine serum (Gibco, Gran Island, NY, USA) and 1% penicillin/streptomycin (Beyotime, Shanghai, China). The human breast epithelial cell line MCF-10A cells were provided via Procell and cultured in DMEM/F12 (Procell) with additional 5% horse serum, 20 ng/mL epidermal growth factor, 0.5 μg/mL hydrocortisone, 10 μg/mL insulin, 1% non-essential amino acids and 1% penicillin/streptomycin. All cells were grown at 37 °C and 5% CO_2_.

The 5-FU-resistant breast cancer cells (MDA-MB-231/5-FU and MDA-MB-453/5-FU) were established using MDA-MB-231 and MDA-MB-453 cells via exposing to the increasing doses of 5-FU (MedChemExpress, Monmouth Junction, NJ, USA), staring at 3.84 μM and ending at 23.0 μM for one month at each step as previous report [24]. The cells were cultured in non-5-FU medium for 15 days prior to each experiment.

### Quantitative reverse transcription polymerase chain reaction (qRT-PCR)

Tissue or cell RNA was isolated using Trizol reagent (Thermo Fisher) based on the procedures as previous report [25]. The RNA was reversely transcribed to cDNA with RevertAid First Strand cDNA Synthesis Kit (Thermo Fisher). Next, the cDNA together with SYBR (Solarbio, Beijing, China) and specific primers (Sangon, Shanghai, China) was used for qRT-PCR on an Applied Biosystems 7500 system (Thermo Fisher). The primers were listed as: circFBXL5 (hsa_circ_0125597) (sense, 5′-CCTGATGATGAATGGGTGAA-3′; antisense, 5′-CACGGAAATCGTTGTTGTTG-3′), FBXL5 (sense, 5′-TTCCGTGGATGAAAAGTCAGA-3′; antisense, 5′-GTTCAGTTGCGGGACCACTA-3′), HMGA2 (sense, 5’-CAGCAAGAACCAACCGGTGA-3’; antisense, 5′-ACTGCAGTGTCTTCTCCCTTC-3′), and miR-216b (sense, 5′-GCCGAGAAATCTCTGCAGGCAA-3′; antisense, 5′-CCAGTGCAGGGTCCGAGGT-3′). U6 (sense, 5′-TCGCTTCGGCAGCACATATAC-3′; antisense, 5′-TATGGAACGCTTCACGAATTTG-3′), or GAPDH (sense, 5′-GAATGGGCAGCCGTTAGGAA-3′; antisense, 5′-AAAAGCATCACCCGGAGGAG-3′) was applied for a reference. The relative RNA abundance was calculated by 2^−ΔΔCt^ method [26].

### Detection of circRNA stability

RNase R (a 3′–5′ exonuclease) and transcription analyses were applied to analyze the circular structure of circRNA as previous report [27]. For RNase R assay, the isolated RNA was incubated with 3 U/μg of RNase R (GeneSeed, Guangzhou, China) for 30 min at 37 °C, and then the circular and linear FBXL5 abundances were detected via qRT-PCR. For transcription analysis, the extracted RNA was reversely transcribed using the Random primers or Oligo(dT)_18_ primers, and then used for qRT-PCR to detect circular and linear FBXL5 abundances.

### Vector and oligonucleotide construct and cell transfection

The circFBXL5 overexpression vector was constructed via inserting the full-length sequence of circFBXL5 into pcDNA3.1(+) CircRNA Mini Vector (Addgene, Watertown, MA, USA) via endonuclease sites Nhe I and Hind III, with the vector alone as negative control (vector). The HMGA2 overexpression vector was constructed via cloning HMGA2 sequence into pcDNA3.1 vector (Thermo Fisher) via endonuclease sites Kpn I and BamH I, with the empty vector as negative control (pcDNA). The siRNA for circFBXL5, negative control of siRNA, miR-216b mimic, negative control of mimic, miR-216b inhibitor, and negative control of inhibitor were formed via GenePharma (Shanghai, China). MDA-MB-231/5-FU and MDA-MB-453/5-FU cells were transfected with the constructed vectors or oligonucleotides using Lipofectamine 3000 (Thermo Fisher) for 24 h. The non-transfected cells were regarded as mock group.

### Cell counting kit-8 (CCK8)

Cell viability was tested via CCK8. MDA-MB-231/5-FU and MDA-MB-453/5-FU cells (1 × 10^4^ cells/well) were added into 96-well plates overnight, and then exposed to different doses of 5-FU for 72 h, followed via incubating with 10 μL of CCK8 solution (Glpbio, Montclair, CA, USA) for 2 h. Subsequently, the absorbance at 450 nm was examined through a microplate reader (BioTek, Winooski, VT, USA). The cell viability was normalized to the control group, and the half maximal inhibitory concentration (IC50) of 5-FU was analyzed according to the viability curve.

### Wound healing analysis

The migration of MDA-MB-231/5-FU and MDA-MB-453/5-FU cells was assessed via wound healing analysis. 2 × 10^5^ MDA-MB-231/5-FU and MDA-MB-453/5-FU cells were added to 12-well plates, and grown to ~ 90% confluence. A straight wound was made via a 200 μL sterile pipette tip. Next, cells were cultured in non-serum medium for 24 h. The width of wound was detected under a microscope (Olympus, Tokyo, Japan). The migrated ability was analyzed according to the wound healing and normalized to the control group.

### Transwell analysis

Cell invasive ability was analyzed via transwell analysis using transwell chamber (BD, Franklin Lakes, NJ, USA) precoated via 100 μL of Matrigel (Solarbio). 5 × 10^5^ MDA-MB-231/5-FU and MDA-MB-453/5-FU cells in medium without serum were added into the upper chamber. 600 μL medium with 10% serum was placed into the lower chambers. After culture for 24 h, cells were stained with 0.1% crystal violet (Solarbio), followed via observation under a microscope (magnification × 100) with 3 random fields.

### Flow cytometry

Cell apoptosis was tested with an Annexin V-FITC apoptosis kit (Beyotime). 2 × 10^5^ MDA-MB-231/5-FU and MDA-MB-453/5-FU cells were seeded in 6-well plates and cultured for 72 h. Subsequently, the cells were collected and then suspended with Annexin V-FITC binding buffer. Then, the cell suspensions were stained with Annexin V-FITC and Propidium Iodide. Finally, the state of cells was analyzed by flow cytometer (Beckman Coulter, Fullerton, CA, USA). The percentage of cells on the upper and lower right quadrants is the apoptotic rate.

### Dual-luciferase reporter analysis and RNA pull-down assay

The targets of circFBXL5 were predicted via starBase (http://starbase.sysu.edu.cn/) [28], and the targets of miR-216b were predicted via DIANA-microT (http://diana.imis.athena-innovation.gr/DianaTools/index.php?r=microT_CDS/index) [29]. The dual-luciferase reporter and RNA pull-down analyses were conducted to analyze the target interaction of miR-216b and circFBXL5 or HMGA2. Briefly, the wild-type (circFBXL5-WT or HMGA2-WT) or mutant luciferase reporter vectors (circFBXL5-MUT or HMGA2-MUT) were generated via inserting the corresponding sequence containing wild-type or mutant miR-216b complementary sites in the pMIR-REPORT vectors (Thermo Fisher) via endonuclease sites Sac I and Spe I. The constructed vectors, control vectors and miR-216b mimic or miR-con were transfected into MDA-MB-231/5-FU and MDA-MB-453/5-FU cells for 24 h. The luciferase activity was detected with dual-luciferase analysis kit (Promega, Madison, WI, USA).

RNA pull-down analysis was performed with a Magnetic RNA–Protein Pull-Down kit (Thermo Fisher). The biotinylated miR-216b mimic (biotin-miR-216b) and negative control (biotin-miR-con) were generated via GenePharma and transfected into MDA-MB-231/5-FU and MDA-MB-453/5-FU cells. After 24 h, cells were lysed and interacted with streptavidin magnetic beads for 6 h. Next, the enrichment abundances of circFBXL5 and HMGA2 were detected via qRT-PCR.

### Western blot

Protein was obtained via using RIPA buffer (Solarbio), and the concentration was examined via a BCA kit (Thermo Fisher). 30 μg protein samples were separated via SDS-PAGE and transferred to nitrocellulose membranes (Bio-Rad, Hercules, CA, USA). 5% non-fat milk was exploited to block the membrane. Subsequently, the membranes were incubated with antibody against HMGA2 (AF4069, 1:1000 dilution, Affinity Biosciences, Cincinnati, OH, USA) overnight and IgG conjugated via HRP (S0001, 1:5000 dilution, Affinity Biosciences) for 2 h. The β-actin (AF7018, 1:5000 dilution, Affinity Biosciences) was regarded as a loading reference. After exposing to ECL reagent (Beyotime), the blots were analyzed by Image Lab software (Bio-Rad).

### Xenograft experiment

The lentiviral vectors carried shRNA for circFBXL5 (sh-circFBXL5) or negative control (sh-con) were generated via GenePharma, and transfected into MDA-MB-231/5-FU cells. The non-transfected cells were regarded as mock group. Female BALB/c nude mice (5-week-old; Charles River, Beijing, China) were arbitrarily divided into mock, sh-con or sh-circ_0006916 groups (n = 6/group). Transfected or non-transfected MDA-MB-231/5-FU cells (5 × 10^6^ cells) were subcutaneously injected into the right flank of nude mice. The tumor size was examined every 7 days. The volume was calculated via length × width^2^/2. All mice were euthanized via 5% isoflurane after cell injection for 28 days. Tumor tissues were weighed and harvested for detection of circFBXL5, miR-216b and HMGA2 expression. This experiment was approved via the Animal Ethical Committee of the First Affiliated Hospital of Zhengzhou University and conducted under the National Institutes of Health.

### Statistical analysis

The experiment was repeated 3 times with 3 replicates, unless otherwise indicated. The linear relationship between circFBXL5 and miR-216b expression in breast cancer tissues was assessed via Pearson correlation test. The data were presented as mean ± SD, and compared via Student’s *t*-test or ANOVA with Tukey test using GraphPad Prism 6 (GraphPad Inc., La Jolla, CA, USA). *P* < 0.05 indicated the significant difference.

## Results

### circFBXL5 abundance is elevated in breast cancer

**Fig. 1:**
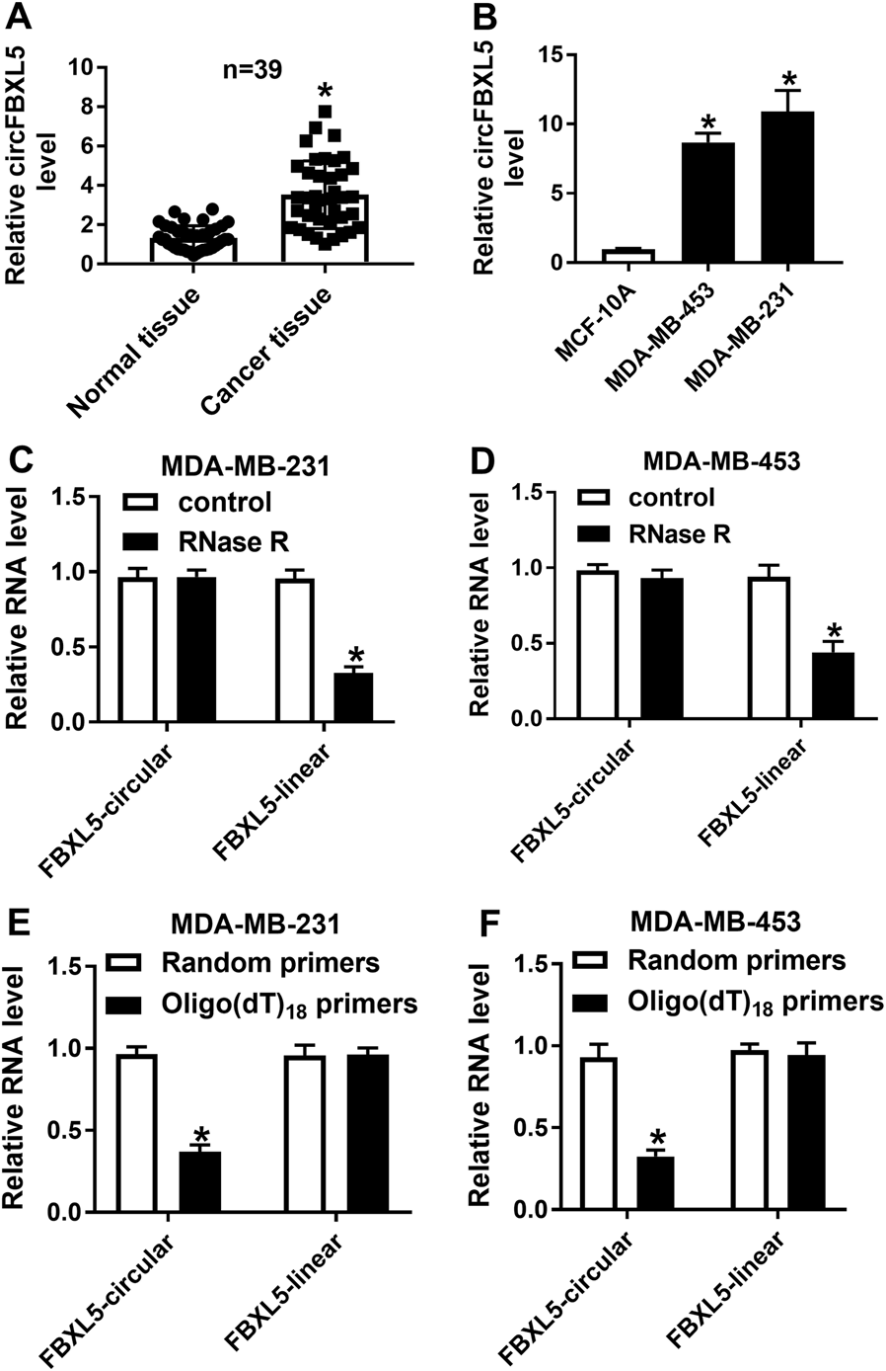
circFBXL5 abundance in breast cancer. **A** circFBXL5 level in breast cancer tissues and normal tissues (n = 39). **B** circFBXL5 expression in breast cancer cell lines (MDA-MB-453 and MDA-MB-231) and normal human breast epithelial cell line MCF-10A. **C**, **D** The circular and linear FBXL5 levels in MDA-MB-231 and MDA-MB-453 cells after treatment of RNase R. **E**, **F** The circular and linear FBXL5 levels in MDA-MB-231 and MDA-MB-453 cells via using the Random primers or Oligo(dT)_18_ primers. ^*^P < 0.05

To explore whether circFBXL5 was implicated in breast cancer progression, we collected 39 paired breast cancer tissues and normal tissues, and measured circFBXL5 expression. As shown in Fig. 1A, circFBXL5 abundance was obvious higher in cancer tissues than normal tissues. The correlation analysis between circFBXL5 expression and clinicopathological characteristics of breast cancer patients showed that high expression of circFBXL5 was associated with the tumor size and TNM stage of breast cancer patients (Table 1). Furthermore, circFBXL5 expression was evidently increased in breast cancer cell lines (MDA-MB-453 and MDA-MB-231) when compared to normal human breast epithelial cell line MCF-10A (Fig. 1B). To identify the circular structure of circFBXL5, the RNA was treated via RNase R. Results displayed that the circFBXL5 was more resistant to RNase R than the linear type (Fig. 1C, D). In addition, the transcription analysis using the Random primers or Oligo(dT)_18_ primers exhibited that the circFBXL5 was almost undetectable when using Oligo(dT)_18_ primers in comparison to Random primers (Fig. 1E, F). These suggested that circFBXL5 had a stable circular structure without poly-A tail. Taken together, circFBXL5 was stably and highly expressed in breast cancer.

### The influence of circFBXL5 on 5-FU resistance, migration, invasion and apoptosis in breast cancer cells

**Fig. 2:**
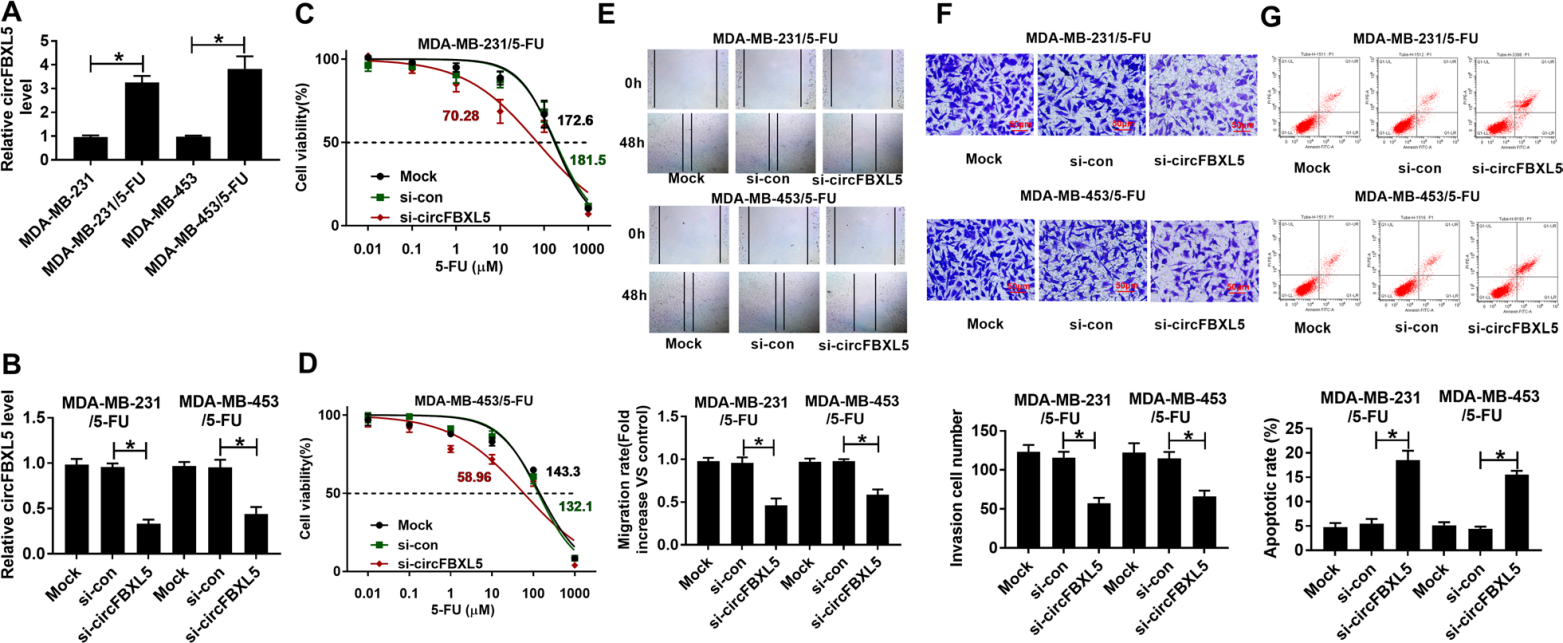
The influence of circFBXL5 on the 5-FU resistance in breast cancer cells. **A** circFBXL5 level in 5-FU-resistant and sensitive MDA-MB-231 and MDA-MB-453 cells. **B** circFBXL5 expression in MDA-MB-231/5-FU and MDA-MB-453/5-FU cells with transfection of si-circFBXL5 or si-con. **C**, **D** Cell viability and IC50 of 5-FU in cells with transfection of si-circFBXL5 or si-con after exposure to various doses of 5-FU for 72 h. **E**, **F** Cell migration and invasion in cells with transfection of si-circFBXL5 or si-con. Bar: 50 μm. **G** Cell apoptosis in cells with transfection of si-circFBXL5 or si-con. Mock: non-transfected group. ^*^P < 0.05

Then, we detected circFBXL5 expression in 5-FU-resistant MDA-MB-231 and MDA-MB-453 cells. The results showed that circFBXL5 abundance was evidently up-regulated in MDA-MB-231/5-FU and MDA-MB-453/5-FU cells when compared to the corresponding normal breast cancer cell lines (Fig. 2A). To analyze the function of circFBXL5 on the 5-FU resistance of breast cancer, circFBXL5 level was knocked down in MDA-MB-231/5-FU and MDA-MB-453/5-FU cells via transfection of si-circFBXL5 (Fig. 2B). Using the CCK8 assay, we measured the 5-FU resistance of breast cancer cells. Results showed that circFBXL5 knockdown markedly decreased the IC50 of 5-FU in MDA-MB-231/5-FU (70.28 vs 181.5 μM) and MDA-MB-453/5-FU (58.96  VS 132.1 μM) cells (Fig. 2C, D). Furthermore, circFBXL5 silence evidently suppressed the abilities of migration and invasion in the two cell lines (Fig. 2E, F). Besides, circFBXL5 interference significantly facilitated the apoptosis of MDA-MB-231/5-FU and MDA-MB-453/5-FU cells (Fig. 2G). These results indicated that circFBXL5 might promote the 5-FU resistance of breast cancer.

### miR-216b is targeted by circFBXL5

**Fig. 3:**
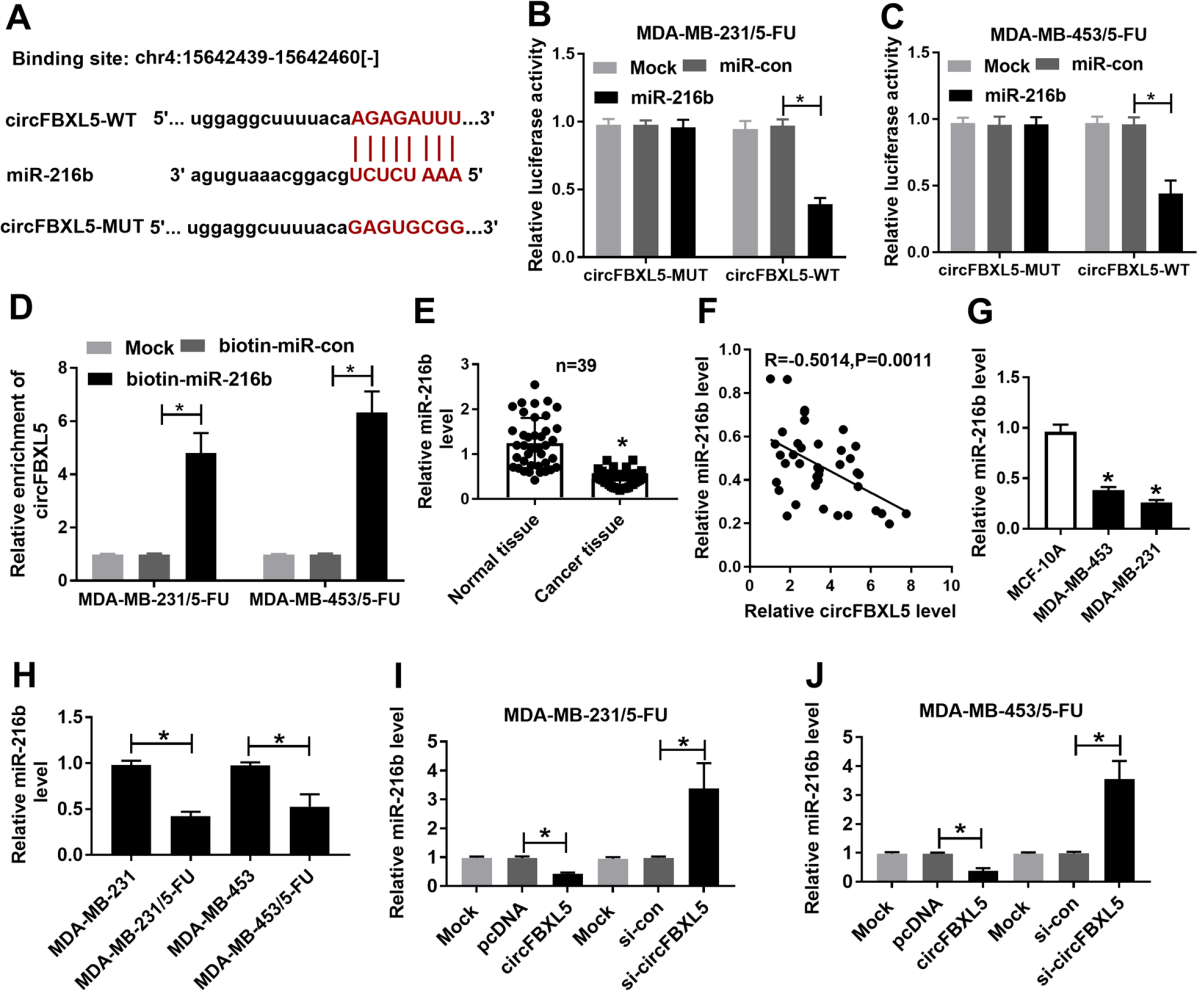
The relationship of circFBXL5 and miR-216b. **A** The binding sequence of circFBXL5 and miR-216b. **B**, **C** Luciferase activity was detected in cells with co-transfection of circFBXL5-WT or circFBXL5-MUT and miR-216b mimic or miR-con. **D** circFBXL5 expression in cells with transfection of biotin-miR-con or biotin-miR-216b after RNA pull-down. **E** miR-216b abundance in breast cancer tissues and normal tissues (n = 39). **F** circFBXL5 and miR-216b expression association in breast cancer tissues. **G** miR-216 abundance in breast cancer cell lines (MDA-MB-453 and MDA-MB-231) and normal human breast epithelial cell line MCF-10A. **H** miR-216 abundance in 5-FU-resistant and sensitive MDA-MB-231 and MDA-MB-453 cells. **I**, **J** miR-216 expression in cells with transfection of vector, circFBXL5 overexpression vector, si-circFBXL5 or si-con. Mock: non-transfected group. ^*^P < 0.05

To explore the regulatory network of circFBXL5, we predicted the targets miRNA for circFBXL5. As a result, miR-216b was found to have binding sequence with circFBXL5 (Fig. 3A). Moreover, we constructed the luciferase reporter vectors of circFBXL5-WT and circFBXL5-MUT, and performed the dual-luciferase reporter analysis. The results exhibited that miR-216b overexpression evidently declined the luciferase activity of circFBXL5-WT vector, but it did not affect the activity of circFBXL5-MUT vector (Fig. 3B, C). In addition, the data of RNA pull-down assay showed that the enrichment of circFBXL5 was markedly increased in the biotin-miR-216b probe (Fig. 3D). Furthermore, miR-216b abundance was examined in breast cancer. Results showed that miR-216b abundance was remarkably declined in breast cancer tissues in comparison to normal tissues (Fig. 3E), and was negatively correlated with circFBXL5 level (R = − 0.5014, *P* = 0.0011) (Fig. 3F). Additionally, we found that miR-216b expression was remarkably downregulated in breast cancer cells and was even lower in 5-FU-resistant breast cancer cells (Fig. 3G, H). Besides, miR-216b abundance was significantly reduced via circFBXL5 overexpression and enhanced via circFBXL5 silence (Fig. 3I, J). These data suggested that miR-216b could be sponged by circFBXL5.

### miR-216b knockdown reverses the influence of circFBXL5 silence on the 5-FU resistance of breast cancer

**Fig. 4:**
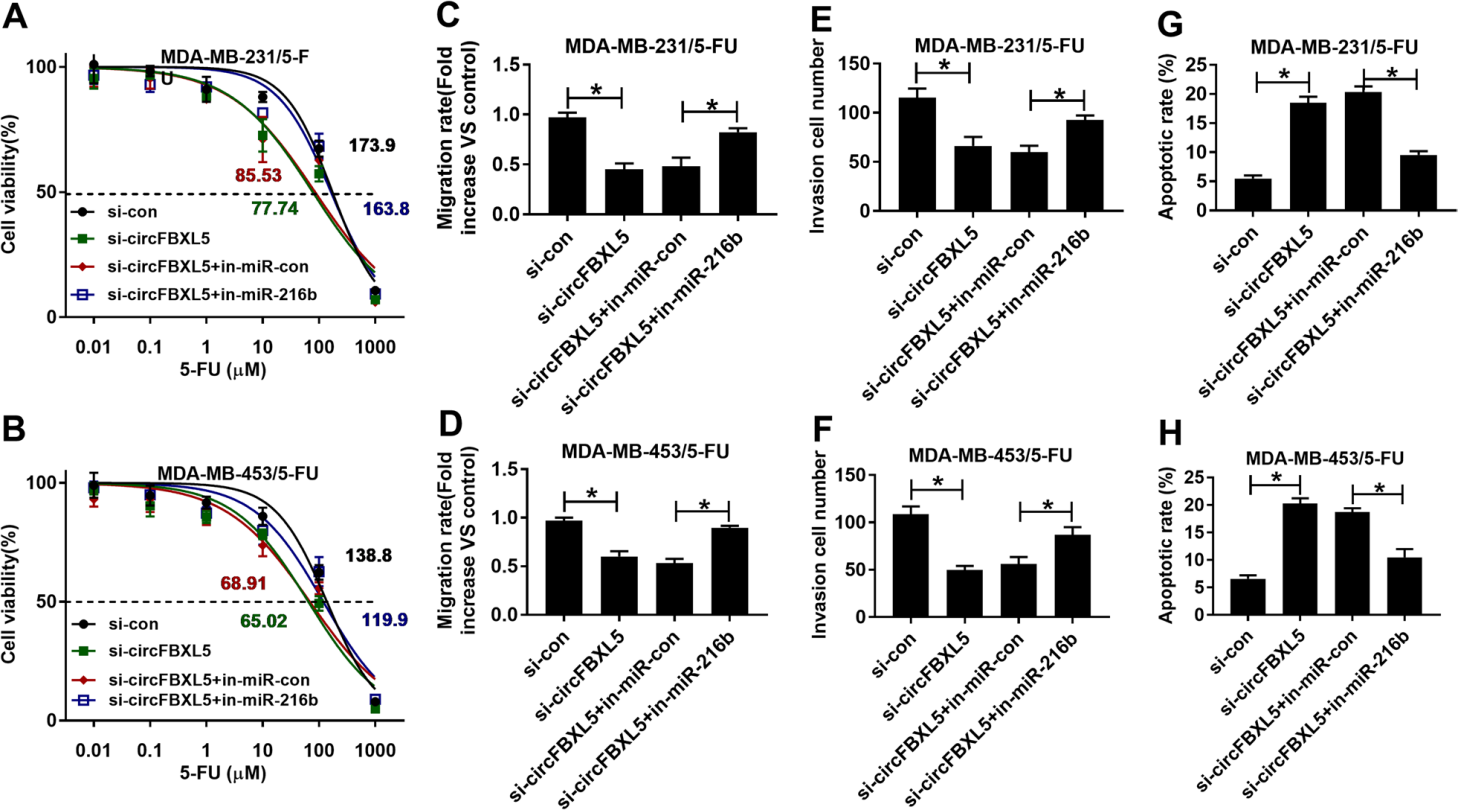
The influence of miR-216b on circFBXL5-mediated 5-FU resistance in breast cancer. Cell viability and IC50 of 5-FU (**A**, **B**), migration (**C**, **D**), invasion (**E**, **F**), and apoptosis (**G**, **H**) in cells with transfection of si-con, si-circFBXL5, si-circFBXL5 + in-miR-con or in-miR-216b. ^*^P < 0.05

To test whether miR-216b was required for circFBXL5-mediated regulation on the 5-FU resistance of breast cancer, MDA-MB-231/5-FU and MDA-MB-453/5-FU cells were transfected with si-con, si-circFBXL5, si-circFBXL5 + in-miR-con or in-miR-216b. Function experiments showed that miR-216b inhibitor reversed the inhibitory effect of circFBXL5 knockdown on the 5-FU resistance in MDA-MB-231/5-FU and MDA-MB-453/5-FU cells (Fig. 4A, B). Additionally, miR-216b inhibitor reversed the suppressive effect of circFBXL5 knockdown on cell migration and invasion (Fig. 4C–F). Besides, the promotion effect of circFBXL5 silencing on the apoptosis of MDA-MB-231/5-FU and MDA-MB-453/5-FU cells also could be abolished by miR-216b inhibitor (Fig. 4G, H). These results suggested that circFBXL5 could regulate 5-FU resistance of breast cancer via modulating miR-216b.

### HMGA2 is targeted via miR-216b

**Fig. 5:**
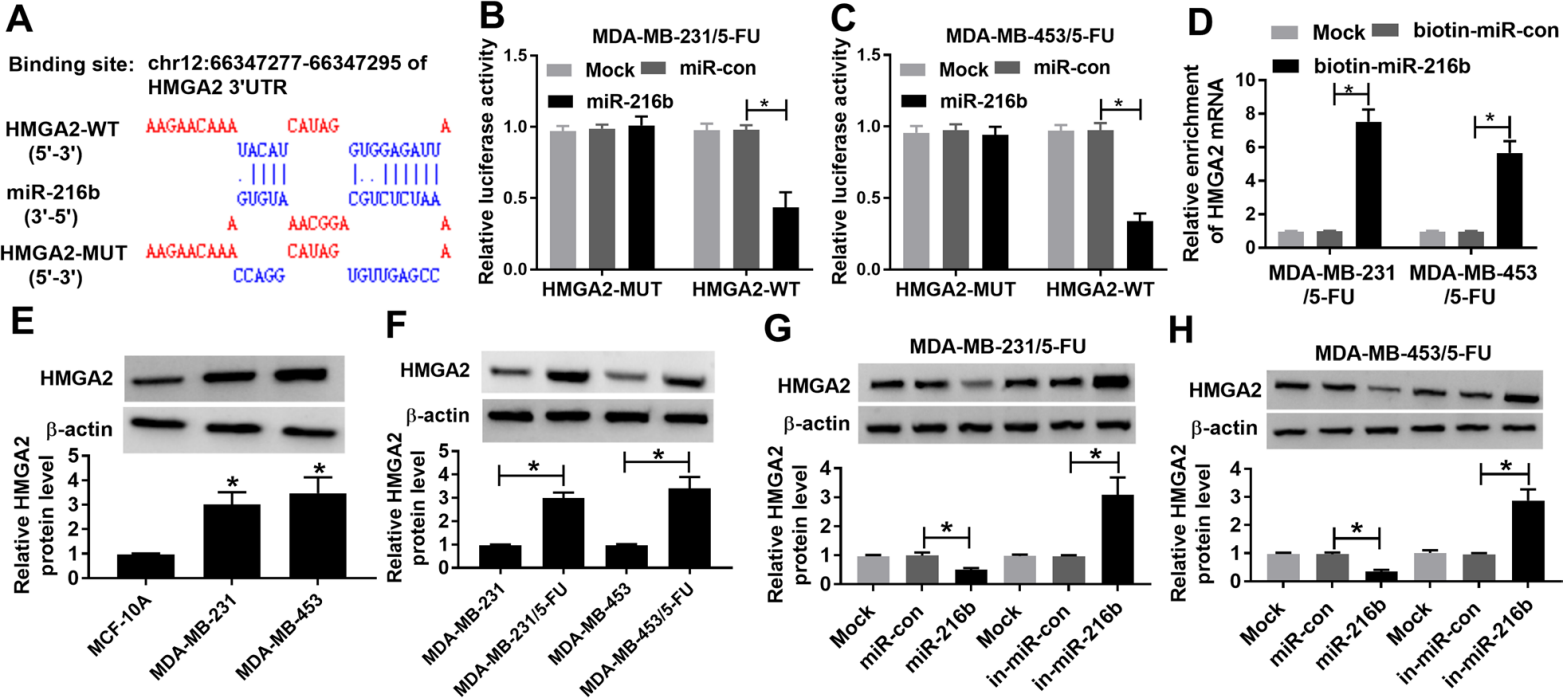
The relationship of miR-216b and HMGA2. **A** The binding sequence of miR-216b and HMGA2. **B**, **C** Luciferase activity was examined in cells with co-transfection of HMGA2-WT or HMGA2-MUT and miR-216b mimic or miR-con. **D** HMGA2 abundance in cells with transfection of biotin-miR-con or biotin-miR-216b after RNA pull-down. **E** HMGA2 protein expression in MDA-MB-453, MDA-MB-231 and MCF-10A cells. **F** HMGA2 abundance in 5-FU-resistant and sensitive MDA-MB-231 and MDA-MB-453 cells. **G**, **H** HMGA2 protein level in cells with transfection of miR-con, miR-216b mimic, in-miR-con or in-miR-216b. Mock: non-transfected group. ^*^P < 0.05

To further explore the regulatory network mediated via circFBXL5 and miR-216b, the targets of miR-216b were predicted. HMGA2 was a predicted target of miR-216b, and their target sequence was displayed in Fig. 5A. To validate this prediction, we constructed the luciferase reporter vectors of HMGA2-WT and HMGA2-MUT. Moreover, the data of dual-luciferase reporter analysis displayed that miR-216b overexpression reduced the luciferase activity of HMGA2-WT vector, but this effect was abolished in HMGA2-MUT vector (Fig. 5B, C). Additionally, RNA pull-down analysis displayed that the enrichment of HMGA2 was significantly increased in the biotin-miR-216b probe (Fig. 5D). Furthermore, HMGA2 protein level was remarkably enhanced in MDA-MB-231 and MDA-MB-453 cells compared with MCF-10A cells, and it was further up-regulated in resistant breast cancer cells (Fig. 5E, F). Besides, we also discovered that HMGA2 protein abundance was evidently declined via miR-216b overexpression and elevated via miR-216b knockdown (Fig. 5G, H). These results indicated that HMGA2 was a target of miR-216b.

### miR-216b suppresses the 5-FU resistance of breast cancer cells via targeting HMGA2

**Fig. 6:**
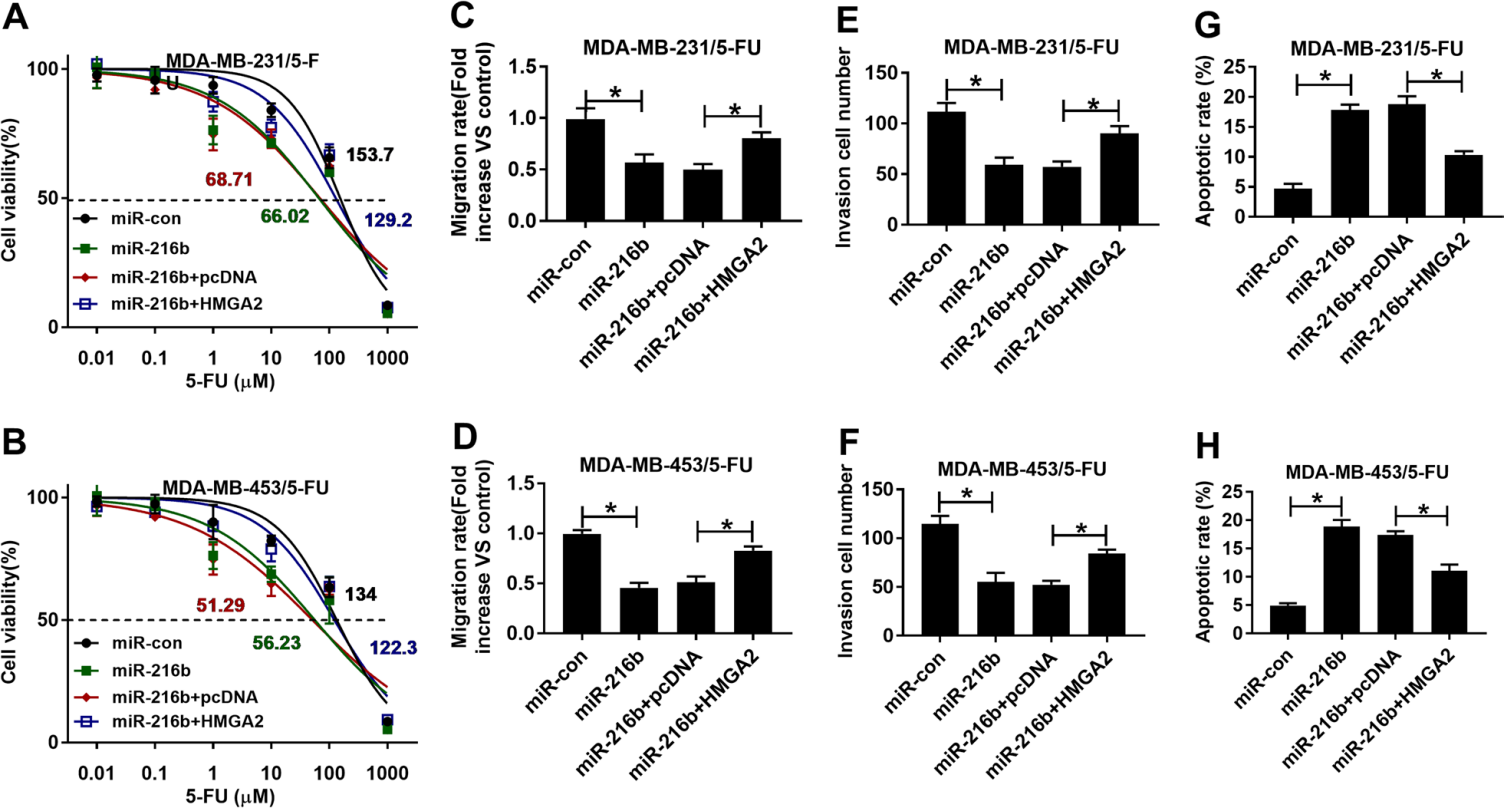
The influence of miR-216b and HMGA2 on the 5-FU resistance of breast cancer cells. Cell viability and IC50 of 5-FU (**A**, **B**), migration (**C**, **D**), invasion (**E**, **F**), and apoptosis (**G**, **H**) in cells with transfection of miR-con, miR-216b mimic, miR-216b mimic + pcDNA or HMGA2 overexpression vector. ^*^P < 0.05

To explore whether miR-216b regulated the 5-FU resistance of breast cancer cells by HMGA2, MDA-MB-231/5-FU and MDA-MB-453/5-FU cells were co-transfected with miR-216b mimic and HMGA2 overexpression vector. Our data showed that miR-216b overexpression inhibited the IC50 of 5-FU in MDA-MB-231/5-FU (66.02 vs 153.7 μM) and MDA-MB-453/5-FU (56.23 vs 134 μM) cells, while these effect could be reversed by overexpressing HMGA2 (129.2 vs 68.71 μM; 122.3 vs 51.29 μM) (Fig. 6A, B). Additionally, miR-216b overexpression evidently inhibited cell migration and invasion, and HMGA2 overexpression also could abolished these effects (Fig. 6C–F). Besides, overexpressed HMGA2 also reversed the increasing effect of miR-216b overexpression on the apoptosis of MDA-MB-231/5-FU and MDA-MB-453/5-FU cells (Fig. 6G, H). These data suggested that miR-216b could inhibit 5-FU resistance of breast cancer via modulating HMGA2.

### circFBXL5 knockdown decreases breast cancer tumor growth in vivo

**Fig. 7:**
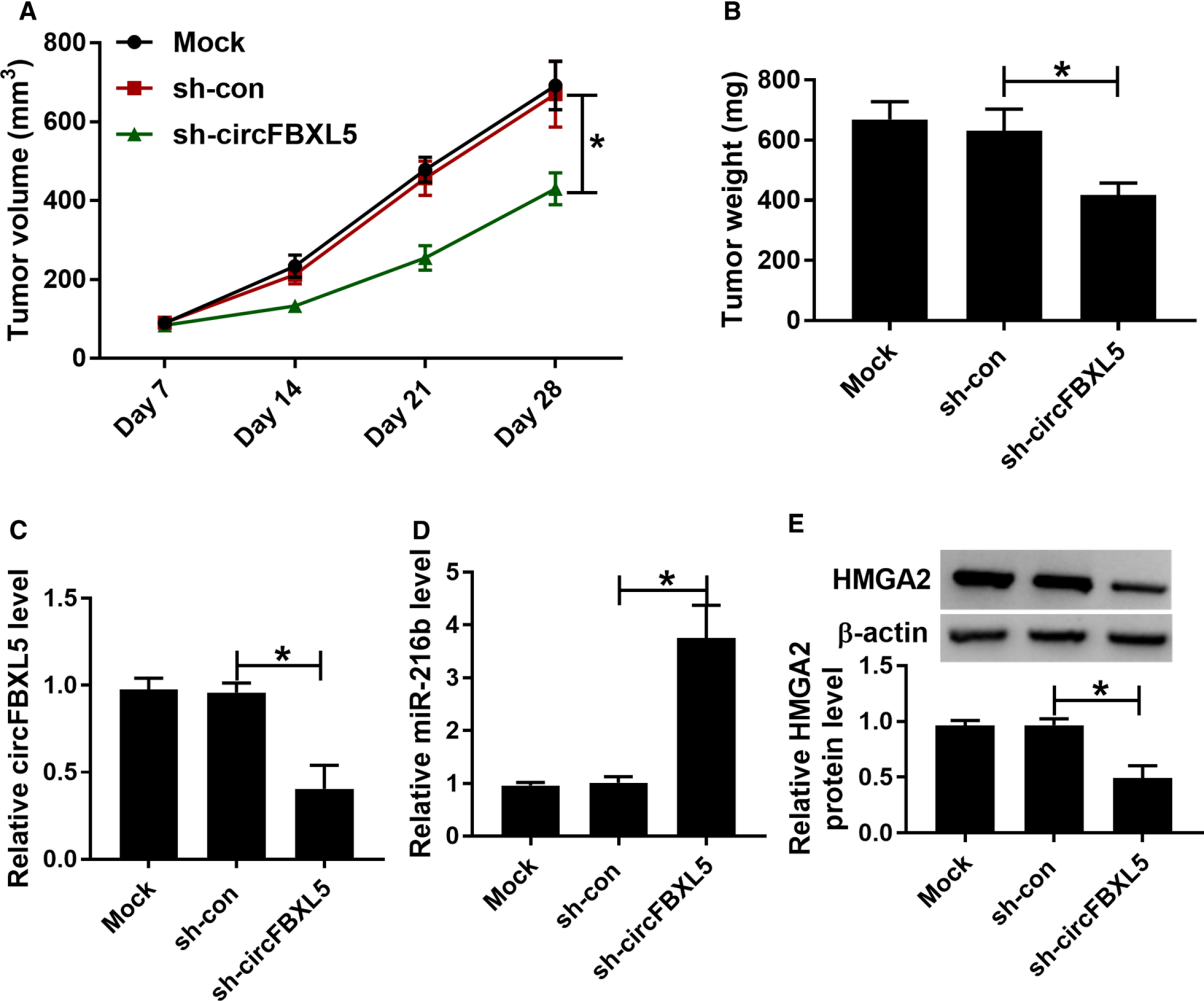
The influence of circFBXL5 on breast cancer tumor growth in vivo. **A** Tumor volume was detected every 7 days. **B** Tumor weight was examined in each group. **C**–**E** circFBXL5, miR-216b and HMGA2 abundances in tumor tissues of each group. ^*^P < 0.05

To test the function of circFBXL5 on breast cancer tumor growth in vivo, MDA-MB-231/5-FU cells transfected with sh-circFBXL5 or sh-con were inoculated into mice. As displayed in Fig. 7A, B, tumor volume and weight were evidently declined in sh-circFBXL5 group compared with sh-con or mock group. Furthermore, circFBXL5, miR-216b and HMGA2 abundances were detected in tumor tissues. Results showed that circFBXL5 expression was evidently reduced while miR-216b expression was significantly enhanced in the sh-circFBXL5 group compared with sh-con or mock group (Fig. 7C–D). Also, HMGB2 protein expression was found to be lowly expressed in the sh-circFBXL5 group (Fig. 7E). These results suggested that circFBXL5 silence reduced 5-FU-resistant breast cancer cell growth.

## Discussion

Breast cancer is a major malignancy with high morbidity in women [30]. Studies have shown that an unhealthy diet may increase the risk of breast cancer recurrence [31]. Magnetic resonance imaging and diffusion weighted imaging have been shown to be effective in differentiating non-lump-like breast lesions [32]. CircRNAs have key roles in the development and treatment of female disorders, including breast cancer [33]. The purpose of this study is to explore a novel insight into understanding the 5-FU resistance to breast cancer. A previous evidence has indicated that circFBXL5 could promote breast cancer development [9]. Nevertheless, little is known about its influence on the 5-FU resistance of breast cancer. Our research confirmed that circFBXL5 knockdown could repress 5-FU resistance in breast cancer cells by inhibiting cell metastasis and promoting apoptosis. Furthermore, we first proposed a potential regulatory network of the circFBXL5/miR-216b/HMGA2 axis.

In this study, we found that circFBXL5 abundance was enhanced in breast cancer, which was also in agreement with former report [9]. This indicated the highly expressed circFBXL5 might play a vital function in breast cancer development. To explore the function of circFBXL5 on the 5-FU resistance of breast cancer, we established the 5-FU resistant cell lines (MDA-MB-231/5-FU and MDA-MB-453/5-FU) as previous report [24]. By exposing to different doses of 5-FU, our results suggested that circFBXL5 knockdown decreased the 5-FU resistance of breast cancer cells. Furthermore, we found that circFBXL5 silence could suppress the 5-FU-resistant cell migration and invasion, while promote apoptosis, which was like its function in the sensitive breast cancer cells [9]. Besides, we used a xenograft model of MDA-MB-231/5-FU cells to further confirm that circFBXL5 knockdown inhibited tumor growth in vivo. Hence, we thought that circFBXL5 might increase 5-FU resistance of breast cancer.

The circRNA/miRNA/mRNA regulatory network is important mechanism for circRNA in cancer development [34]. A previous study has indicated that circFBXL5 could sponge miR-660 to regulate serine/arginine-rich splicing factor 6 (SRSF6) [9]. In this, our research aimed to explore additional regulatory network mediated via circFBXL5. Here we first identified miR-216b could be sponged by circFBXL5. Jana et al. reported that miR-216b inhibited cell migration and invasion of breast cancer by regulating SDCBP [16]. Zheng et al. suggested that miR-216b could promote breast cancer cell apoptosis via regulating P2X7 receptor (P2X7R) [35]. Similarly, our study validated that miR-216b overexpression suppressed migration and invasion and triggered apoptosis of 5-FU-resistant breast cancer cells. Furthermore, we found that miR-216b could inhibit 5-FU resistance in breast cancer, which was also in agreement with that in hepatocellular carcinoma cells [15]. Furthermore, miR-216b knockdown abated the influence of circFBXL5 silence on the 5-FU resistance of breast cancer cells, indicating that circFBXL5 regulated the 5-FU resistance of breast cancer via sponging miR-216b.

Having established the circFBXL5/miR-216b axis, we further confirmed miR-216b could target HMGA2. The previous studies reported that HMGA2 could contribute to breast cancer cell metastasis, while inhibit apoptosis [36, 37]. In addition, increasing evidences had suggested that HMGA2 could facilitate 5-FU resistance in many cancers, like hepatoma cancer, colorectal cancer and breast cancer [21–23, 38, 39]. The rescue experiments confirmed that HMGA2 overexpression reversed the suppressive influence of miR-216b on the 5-FU resistance of breast cancer. Collectively, circFBXL5 could target HMGA2 to regulate 5-FU resistance using miR-216b as a crosstalk.

**Fig. 8:**
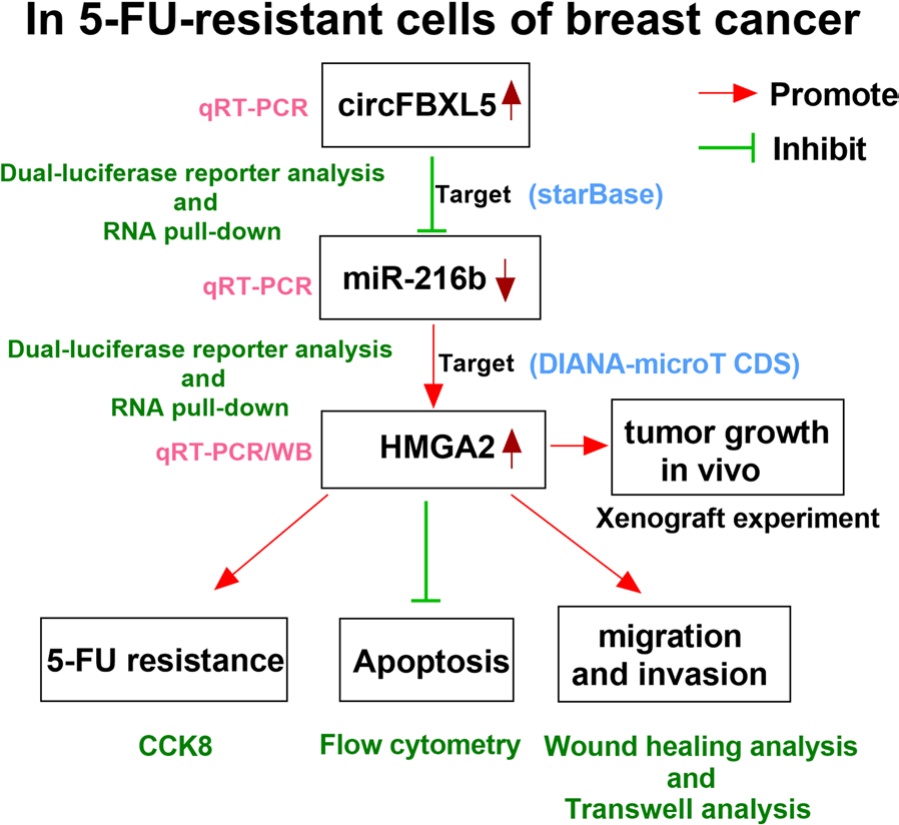
The flowchart and main ideas of this study. CircFBXL5 could sponge miR-216b to regulate HMGA2, thereby promoting the 5-FU resistance of breast cancer by regulating cell migration, invasion and apoptosis

In conclusion, our data showed that circFBXL5 might promote the 5-FU resistance of breast cancer by regulating cell migration, invasion and apoptosis, possibly by regulating the miR-216b/HMGA2 axis (Fig. 8). This work proposes a new insight into the mechanism of the 5-FU resistance for breast cancer and indicates a novel target for breast cancer treatment.

## Data Availability

Not applicable.
